# Salting‐out extraction of recombinant κ‐carrageenase and phage T7 released from *Escherichia coli* cells

**DOI:** 10.1002/elsc.202200125

**Published:** 2023-05-17

**Authors:** Da Chen, Yue‐Sheng Dong, Yong‐Ming Bao, Zhi‐Long Xiu

**Affiliations:** ^1^ School of Bioengineering Dalian University of Technology Dalian Liaoning PR China

**Keywords:** κ‐carrageenase, cell disruption, *E. coli*, phage, salting‐out extraction

## Abstract

Traditional technology of cell disruption has become one of the bottlenecks restricting the industrialization of genetic engineering products due to its high cost and low efficiency. In this study, a novel bioprocess of phage lysis coupled with salting‐out extraction (SOE) was evaluated. The lysis effect of T7 phage on genetically engineered *Escherichia coli* expressing κ‐carrageenase was investigated at different multiplicity of infection (MOI), meanwhile the phage and enzyme released into the lysate were separated by SOE. It was found that T7 phage could lyse 99.9% of host cells at MOI = 1 and release more than 90.0% of enzyme within 90 min. After phage lysis, 87.1% of T7 phage and 71.2% of κ‐carrageenase could be distributed at the middle phase and the bottom phase, respectively, in the SOE system composed of 16% ammonium sulfate and 20% ethyl acetate (w/w). Furthermore, κ‐carrageenase in the bottom phase could be salted out by ammonium sulfate with a yield of 40.1%. Phage lysis exhibits some advantages, such as mild operation conditions and low cost. While SOE can efficiently separate phage and intracellular products. Therefore, phage lysis coupled with SOE is expected to become a viable alternative to the classical cell disruption and intracellular product recovery.

**Abbreviations**
*E. coli*
: *Escherichia coli*
MOI: multiplicity of infection
SOE: salting‐out extraction

## 1 INTRODUCTION

Traditional cell disruption methods include mechanical, chemical, and biological techniques [1, 2, 3], all of which have the disadvantages of high cost and low efficiency. In addition, the subsequent separation and purification of target proteins is difficult [4, 5, 6]. The disruption of *Escherichia coli* cells can be considered as a representative of this process, because *E. coli* is the most commonly used host bacterium for genetic expression of engineered heterologous proteins [7, 8]. These proteins are often in the form of intracellular products [9].

κ‐carrageenase is a polysaccharide hydrolase characterized by high selectivity and good thermal stability, and is widely used in the preparation of κ‐carrageenan oligosaccharides [10]. However, κ‐carrageenase produced by wild strains has low activity and poor stability, and its purification process is also complex [11]. Therefore, genetic engineering for the heterologous expression of κ‐carrageenase has become a common method for the mass production of this enzyme. For example, the κ‐carrageenase gene can be efficiently expressed in *E. coli* cells, and then overproduced as an intracellular enzyme after a low‐temperature induction [12]. Therefore, cell disruption is required to release it.

Bacteriophages [13] are viruses that infect bacteria, including actinomycetes and spirochetes, and fungi. In the treatment of infections caused by multidrug‐resistant bacteria [14, 15, 16], bacteriophage therapy offers a number of advantages, namely the variety of phages, strong specificity (e.g., the T7 phage is a specific phage of *E. coli* [17], and low toxicity. The action mode of phage therapy is lysis of the host bacterial cells at the end of the proliferation stage [18], and this process can be viewed as a potential method of cell disruption. Compared to conventional cell disruption methods, phage‐mediated lysis has some advantages, for example, mild operating conditions, ease of scale up, and low cost. The lysed host cells are relatively complete without small cell debris [19], which is conducive to subsequent separation and purification. Furthermore, many progeny phages can be generated while obtaining intracellular products. On the other hand, bacteriophage infection may cause economic loss in industrial production [20]. Therefore, it is necessary to avoid bacteriophage infection during microbial fermentation, that is, phage lysis in another workshop as well as strict sterilization of the air system and relevant equipments and pipes. Of course, the optimal utilization of both bacteriophages and intracellular products ultimately depends on their effective separation. To date, no studies about the phage‐mediated lysis for intracellular products are reported. An effective separation of both bacteriophages and intracellular products needs to investigate based on phage‐mediated lysis.

Salting‐out extraction (SOE) [21, 22] is a separation and purification technique that integrates solid‐liquid separation, crude separation, as well as concentration, and can be used for the separation and purification of biological macromolecules, such as proteins [23], enzymes [24], viruses [25], and nucleic acids [26]. Our team developed a two‐step SOE method to isolate, purify, and concentrate the phage of *Klebsiella pneumoniae* at the interface between the top and bottom phases in the SOE at the second step [27]. Recently, a one‐step SOE was investigated for the separation and purification of *Acinetobacter baumannii* phage, which was also concentrated at the two‐phase interface [28]. Based on the previous works, SOE is expected to separate intracellular enzyme and phage from the phage lysate by means of the different distribution behaviors of κ‐carrageenase and phage in the SOE.

In the present study, T7 phage was used as a cell disruption tool to explore the feasibility of both phage lysis of genetically engineered *E. coli* cells and release of intracellular κ‐carrageenase. A SOE system was then developed to separate the intracellular products, κ‐carrageenase, from the phage lysate of *E. coli* cells. κ‐carrageenase was finally obtained by salting out. A novel integrated bioprocess for cell disruption and intracellular product recovery would be set up with the aid of phage lysis and SOE.

**PRACTICAL APPLICATION**

The downstream processing of intracellular products needs cell disruption and separation with high efficiency under mild conditions. We demonstrated that phages can be used as a cell disruption tool to lyse host cells in a short time without the activity loss of intracellular enzyme. Additionally, salting‐out extraction (SOE) can be used to distribute progeny phages and κ‐carrageenase to the middle and bottom phases with recovery of 87.1% and 40.1%, respectively. Phage lysis coupled SOE would provide a novel and valuable tool for cell disruption and recovery of intracellular products.

## 2 MATERIALS AND METHODS

### 2.1 Chemicals and reagents

κ‐Carrageenan were purchased from Aladdin (Shanghai, China). All other reagents, for example, potassium sodium tartrate (NaKC_4_H_4_O_6_), 3,5‐dinitrosalicylic acid, phenol, disodium hydrogen phosphate dodecahydrate (Na_2_HPO_4_·12H_2_O), sodium dihydrogen phosphate dehydrate (NaH_2_PO_4_·2H_2_O), sodium sulfite (Na_2_SO_3_), ammonium sulfate ((NH_4_)_2_SO_4_), dipotassium hydrogen phosphate (K_2_HPO_4_), potassium dihydrogen phosphate (KH_2_PO_4_), trisodium citrate dihydrate (Na_3_C_6_H_5_O_7_·2H_2_O), ammonium citrate ((NH_4_)_3_C_6_H_5_O_7_), ethyl acetate, n‐octanol, tert‐butanol, n‐amyl alcohol were analytical reagent grade and purchased from Kermel Chemical Reagent Co. Ltd. (Tianjin, China).

### 2.2 Bacterial strains, phages, and culture conditions

#### 2.2.1 Bacterial strains and phages

The genetically engineered *E. coli* C2, constructed to express κ‐carrageenase by the group of Prof. Yongming Bao [12], was stocked in 50% (v/v) glycerol solution at −80°C. The T7 phage was the gift of the State Key Laboratory of Microbial Technology of Shandong University (Prof. Youming Zhang) and stocked in 60% (v/v) glycerol broth at −80°C.

#### 2.2.2 Culture condition of genetically engineered *E. coli* C2

The genetically engineered *E. coli* C2 was grown as seed solution in Lysogeny Broth (LB) medium supplemented with kanamycin (50 μg/mL) in shaking bed at 200 r/min and 37°C for 14 h. After then, 500 μL seed solution were inoculated in 50 mL LB medium containing 50 μg/mL kanamycin at 37°C and 200 r/min for 3 h, and the expression of the target genes was subsequently induced by 50 μL IPTG (final concentration 1mM) at 19°C and 150 r/min for 12 h.

#### 2.2.3 Propagation of T7 phage

The seed solution was cultured with 1% inoculum in 50 mL LB in shaking bed at 200 r/min and 37°C for 3 h. At the end, T7 phage was added at a multiplicity of infection (MOI) of 1, and continued to culture for 2 h.

### 2.3 Phage lysis

**FIGURE 1:**
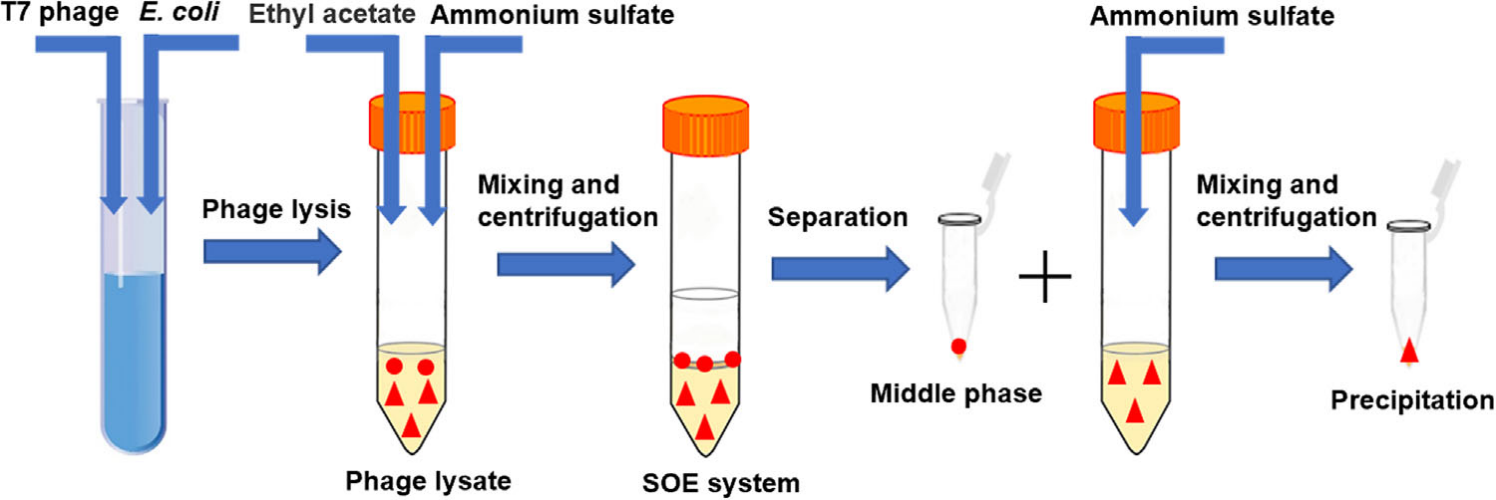
Schematic diagram of phage lysis coupled with SOE of T7 phage (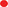) and κ‐carragenase (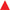).

All manipulations were performed under sterile conditions. *E. coli* C2 induced by IPTG was mixed with T7 phage stock in sterile shaking tubes at MOI of 50, 25, 10, 1, 0.1, respectively, and phage lysis was subsequently performed in shaking bed at 37 °C and 200 r/min for 30–150 min (see Figure 1). After the lysis, the culture broth was termed as phage lysate, which was composed of T7, κ‐carrageenase, and host cells and cell debris, stored at 4 °C before use. By measuring the phage titer, κ‐carrageenase activity and viable cell counts of each sample, the intracellular product release rate [29] and cell disruption rate [30] were calculated to evaluate the lytic effect of T7 phage on genetically engineered *E. coli* C2. The cell disruption rate (%) and the κ‐carrageenase release rate (%) was calculated based on Equations (1) and (2), respectively.

(1) $$\mathrm{Cell}\;\mathrm{disruption}\;\mathrm{rate}\;\left(\%\right)=\left(1-\frac{N_{t}}{N_{0}}\right)\times 100\%$$

where N_0_ and N_t_ are the viable cell counts of *E. coli* before and after phage lysis, respectively.

(2) $$\kappa -\mathrm{carragenase}\;\mathrm{release}\;\mathrm{rate}\;(\%)=\frac{U_{t}}{U_{0}}\times 100\%$$

where U_0_ and U_t_ are total κ‐carrageenase activity after ultrasonic disruption and phage lysis, respectively.

### 2.4 Separation of phage and κ‐carrageenase

All manipulations were performed under non‐sterile conditions, several SOE systems were chosen by combining salts (e.g., ammonium sulfate and dipotassium hydrogen phosphate) with organic solvents (esters and alcohols). The total mass of the SOE systems was 10 g and SOE was prepared in non‐sterile 50 mL centrifugal tubes (see Figure 1), and the whole process was carried out in an ice bath. Firstly, the salt powder was slowly added to the phage lysate, and the salt was completely dissolved by gentle stirring. Then the pre‐cooled organic solvent was slowly added, and the mixture was thoroughly mixed. After standing at 4°C for 30 min, the top and bottom phases were separated by the centrifugation at 2000 g and 4°C for 10 min, and the aggregates in the interphase between the top and bottom phase were completely collected and dissolved in 20 mM PBS buffer at pH = 8. After SOE, κ‐carrageenase was dialyzed using a dialysis bag with a molecular weight cut‐off (MWCO) of 3500 Da in 20 mM PBS buffer (pH = 8) at 4°C for 24 h. During dialysis, the dialysate was changed one time. Phage titer, κ‐carrageenase activity, protein concentration and OD_600_ in each phase were determined. The recovery rates of phage and κ‐carrageenase as well as the removal rates of cells/cell debris and proteins in bottom phase were used as evaluation criteria to determine the optimal system. The recovery rate of phage in SOE (Yp) was calculated by the phage titer of each phase after SOE divided by the phage titer in the crude phage lysate using Equation (3). The recovery rate of κ‐carrageenase in SOE (Y_c_) was calculated by the total κ‐carrageenase activity of each phase after SOE divided by the total κ‐carrageenase activity in the crude phage lysate using Equation (4).

(3) $$Y_{p}\left(\%\right)=\frac{C_{t}V_{t}}{C_{0}V_{0}}\times 100\%$$

(4) $$Y_{c}\;\left(\%\right)\;=\frac{U_{t}V_{t}}{U_{0}V_{0}}\times 100\%$$

where V, C, and U are volume, phage titer, and κ‐carrageenase activity, respectively. In SOE, the removal rate of proteins/cells in phage lysate is defined as Equation (5).

(5) $$R_{i}\;\left(\%\right)=100-\left(\frac{C_{i,B}\;V_{i,B}}{C_{i,0}V_{i,0}}\right)\times 100\%$$

where “i” represents total proteins or cell debris.

### 2.5 Salting out of κ‐carrageenase

The ammonium sulfate powder was slowly added to phage lysate, and the saturation of ammonium sulfate was set to 20%, 30%, 40%, 50%, 60%, 70%, 80%, and 90%. After stirring slowly in an ice bath until the salt is completely dissolved, the salting‐out solution was placed in the refrigerator at 4°C overnight and then centrifugated at 8000 g and 4°C for 20 min. The precipitate was completely collected and dissolved in 20 mM PBS buffer (pH = 8) [31, 32], and κ‐carrageenase was then dialyzed using a dialysis bag with a MWCO of 3500 Da in 20 mM PBS buffer (pH = 8) at 4°C for 24 h. During dialysis, the dialysate was changed one time. The κ‐carrageenase distributed into the salt‐rich bottom phase after SOE would be salted out by adding ammonium sulfate according to the above optimal condition.

### 2.6 Analytical techniques

#### 2.6.1 Determination of the phage titer

Phage titer was determined by phage spot assay. 700 μL genetically engineered *E. coli* C2 cultured to exponential growth phase was poured onto on the whole plate, and the excess liquid was removed. After plates were completely dried for 10–30 min, 5 μL of each phage samples with gradient dilutions were spotted onto the bacterial overlay. Subsequently, they were dried for 15 min and incubated for 3–5 h at 37°C in a constant temperature incubator. Phage titer was calculated based on Equation (6).

(6) $$\begin{array}{ccc} \mathrm{Phage}\;\mathrm{titer}\left(\mathrm{PFU}/\mathrm{mL}\right) & = & \mathrm{Plaque}\mathrm{number}\\ & & \times \;\mathrm{dilution}\;\mathrm{multiple}\times 200 \end{array}$$

#### 2.6.2 Determination of the κ‐carrageenase activity

The κ‐carrageenase activity was determined by 3,5‐dinitrosalicylic acid (DNS) method [33]. 500 μL 0.5% κ‐carrageenan (dissolved in 20 mM PBS buffer, pH 8.0) was incubated with 500 μL sample containing κ‐carrageenase in a 45°C water bath for 30 min. After the reaction, 500 μL reaction solution was added to 500 μL DNS reagent in a boiling water bath for 5 min. The absorbance (OD_520_) was then measured after the samples were cooled to room temperature. The control group was inactivated enzyme solution. κ‐carrageenase activity was calculated by using the formula of standard curve constructed with D‐galactose. One unit of κ‐carrageenase activity (U) was defined as the amount of κ‐carrageenase required to produce 1 μmol of reducing sugar per minute under optimal reaction conditions (pH 8.0, 45°C for 30 min).

#### 2.6.3 Determination of viable cell counts

Viable cell counts were determined by agar plate dilution method. The bacterial suspension before and after phage lysis was gradient diluted with 0.9 % normal saline, and 50 μL of the gradient diluted bacterial suspension was evenly spread on LB solid medium. Until they were allowed to dry for 10 min and incubated for 24 h in constant temperature incubator at 37°C. Viable cell counts were calculated according to Equation (7).

(7) $$\begin{array}{ccc} & & \mathrm{Cell}\;\mathrm{counts}\left(\mathrm{CFU}/\mathrm{mL}\right)\\ & & \;=\mathrm{Colony}\;\mathrm{number}\;\mathrm{dilution}\;\mathrm{factor}\times \;20 \end{array}$$

#### 2.6.4 Host cells and suspensions measurement

The concentrations of host cells and suspensions (e.g., cell debris) in each sample were determined by spectrophotometer at 600 nm with pure water as the contrast. The absorbance values of bacterial solution before and after phage lysis were measured, and the host cells and cell debris in each phase of SOE were characterized.

#### 2.6.5 Determination of the protein concentration

Protein concentration was determined by BCA assay. 20 μL of the sample was mixed with 200 μL of BCA reagent in a 96‐well plate, incubated in a constant temperature incubator at 37°C for 30 min, and then the absorbance (OD_562_) was measured. The protein concentration of each sample was calculated by using the standard curve constructed with standard Bovine Serum Albumin.

### 2.7 Statistical analysis

All data were presented in mean ± standard deviation of three independent experiments. Statistical analysis was accomplished by SPSS (version 18.0, Chicago, USA). *P* < 0.05 indicates significant difference between the two groups of data, otherwise indicates insignificant difference.

## 3 AND DISCUSSION

### 3.1 The optimal conditions for phage lysis

**FIGURE 2:**
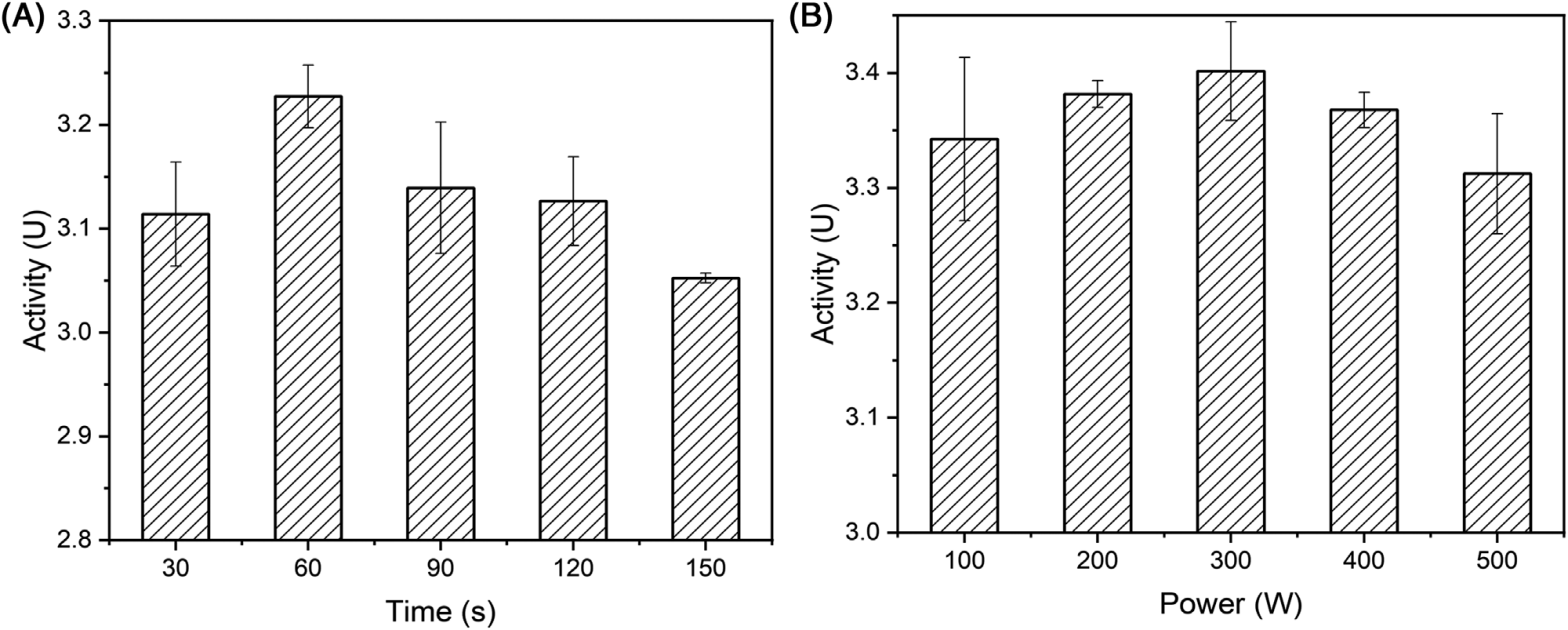
Effects of ultrasound treatment on κ‐carrageenase activity under different conditions. (A) Different time and 300 W; (B) different powers and 60 s.

Soluble expression of κ‐carrageenase was detected in the *E. coli* C2 cells, and the maximum enzyme activity was obtained by ultrasonication. The experimental results are shown in Figure 2. At 300 W, the maximum total κ‐carrageenase activity was obtained after 60 s of ultrasonication. Therefore, ultrasonic power of 300 W and treatment time of 60 s were set as the optimal conditions to achieve the maximum κ‐carrageenase activity, which was considered as the baseline activity.

**FIGURE 3:**
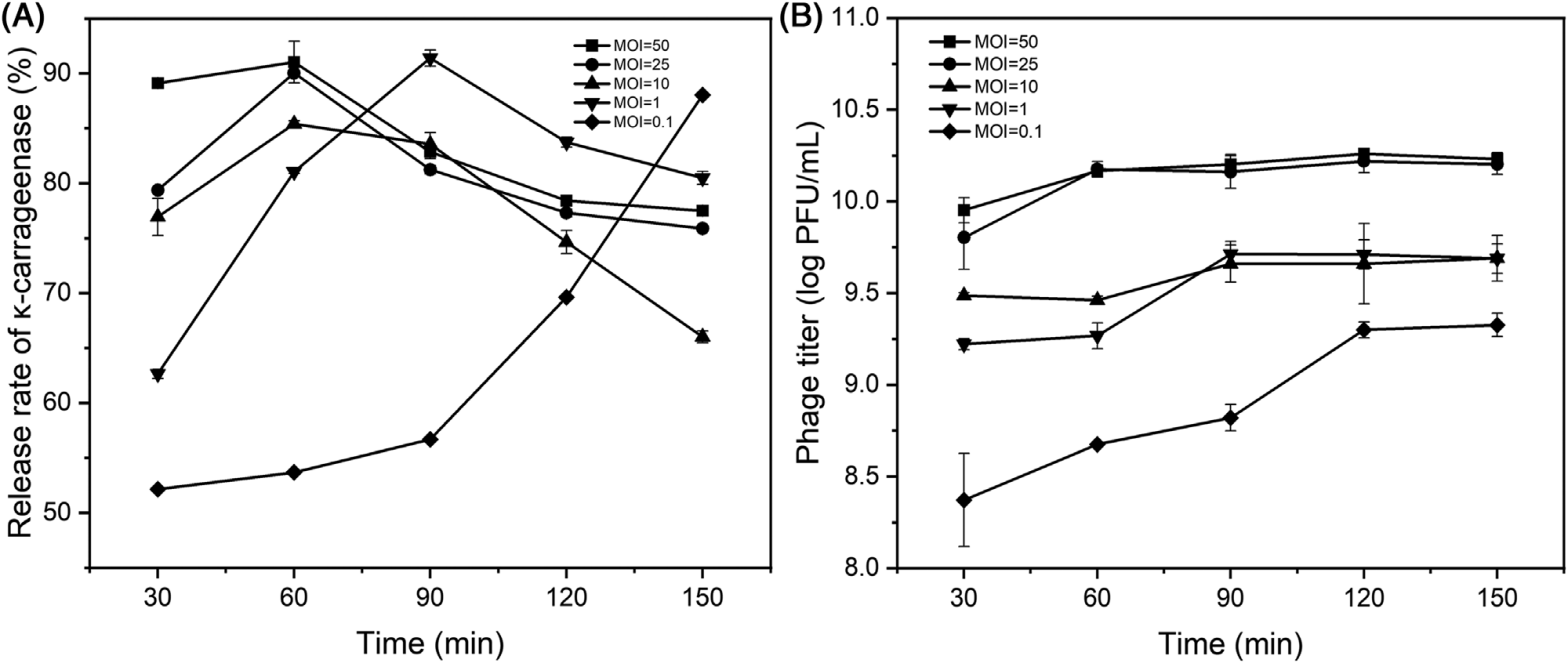
Release rates of κ‐carrageenase (A) and phage (B) at different multiplicity of infection (MOI) and time.

Subsequently, T7 was used to lyse *E. coli* cells. As shown in Figure 3A, the release rates of κ‐carrageenase were 91.0%, 90.0%, and 85.4% at 60 min and MOI of 50, 25, and 10, respectively. When the incubation time increased, the release rate of κ‐carrageenase firstly increased and then decreased. The maximum release rate of κ‐carrageenase was 91.4% and 88.04% at incubation time of 90 and 150 min at MOI of 1 and 0.1, respectively. MOI was commonly defined as the ratio of phages to bacteria in a culture. With the increase of the MOI, the lysis time of the phage decreased, which was consistent with the results reported by Konopacki et al [34]. However, the selection of MOI should not be too large, otherwise, leading to the death of the host cells and low phage replication and amplification.

A large number of progeny phages can also be obtained using phage lysis as a tool for cell disruption. The experimental results are shown in Figure 3B. The greater the MOI, the higher the titer of the progeny phage in the lysate. The time at the maximum titer of progeny phage was corresponding to the time at the maximum release rate of κ‐carrageenase, indicating synchronous release of progeny phage and target enzyme at the same time. For example, when MOI = 1, both the maximum release rate of κ‐carrageenase and highest titer of phage were achieved at 90 min.

**FIGURE 4:**
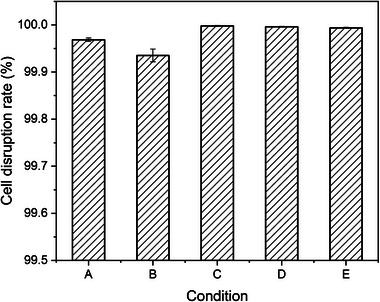
Cell disruption rate by phage lysis at different conditions. (A) Phage lysis for 60 min at MOI = 50; (B) Phage lysis for 60 min at MOI = 25; (C) Phage lysis for 60 min at MOI = 10; (D) Phage lysis for 90 min at MOI = 1; (E) Phage lysis for 150 min at MOI = 0.1.

From the view of cell disruption, the optimal conditions for phage lysis could also be obtained at different MOIs and incubation time. Based on the release rate of the intracellular products, five lysis conditions were selected: 60 min at MOI = 50, 25, and 10; 90 min at MOI = 1; and 150 min at MOI = 0.1. As can be seen in Figure 4, the cell disruption rate could reach more than 99.9% under five different conditions, and the highest could reach 99.99%. According to Equation (1), the viable cell counts of *E. coli* were reduced by 4 orders of magnitude by phage treatment. Because the bacterial cells could develop resistance to phage [35], it is very difficult for cell disruption rate to reach 100%. Considering together the cost, time, and cell disruption, the optimal conditions for phage lysis were determined as 90 min at MOI = 1.

### 3.2 Selection of SOE system

**FIGURE 5:**
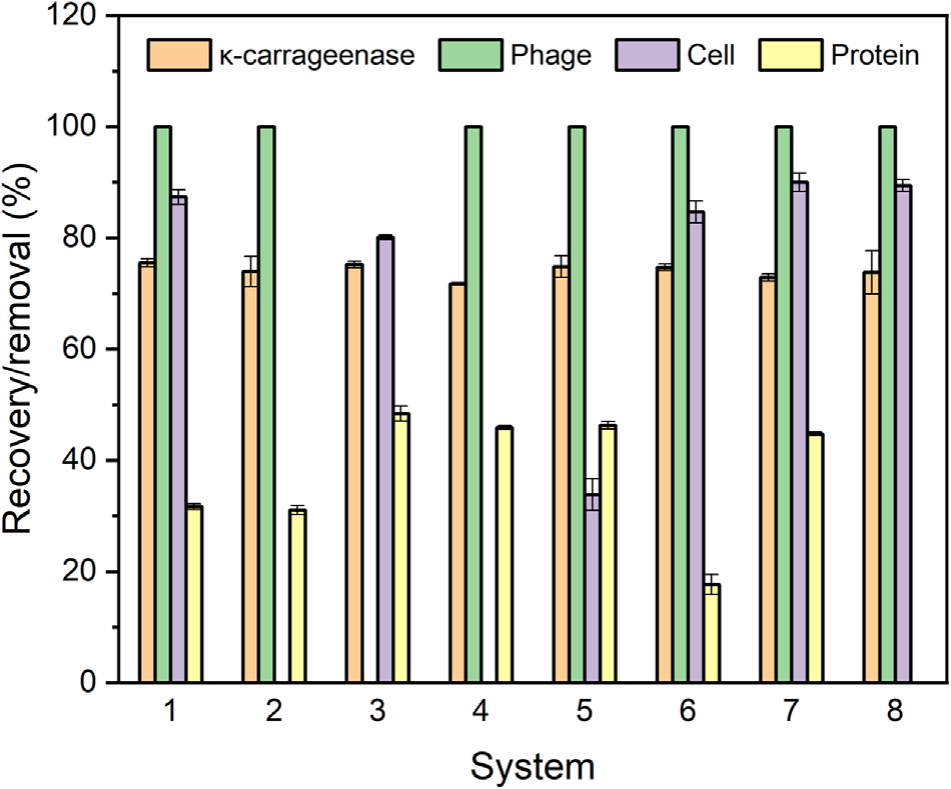
Recovery rates of κ‐carrageenase and phage, removal rates of cells/cell debris and proteins in different SOE systems. System 1: ethyl acetate/ammonium sulfate; 2: n‐octanol/ammonium sulfate; 3: tert‐butyl alcohol/ammonium sulfate; 4: n‐decyl alcohol/ammonium sulfate; 5: n‐amyl alcohol/ammonium sulfate; 6: ethyl acetate/trisodium citrate dihydrate; 7: ethyl acetate/ammonium citrate; 8: ethyl acetate/potassium dihydrogen phosphate: dipotassium hydrogen phosphate (7:15), pH = 7.5.

The effects of different organic solvents and salts on κ‐carrageenase and phage activities were evaluated, the results are shown in Figure 5. In the eight SOE systems composed of 10% different kinds of salt, 20% different kinds of organic solvent and 70% phage lysate (w/w), phage and κ‐carrageenase were mainly distributed in the salt‐rich bottom phase. The recovery rates of κ‐carrageenase in all the 8 systems were 75.0% approximately. Among the different salts, ammonium sulfate is preferred for SOE, because it is usually used as salting‐out agent to concentrate the proteins [31]. Except System 3(tert‐butanol/ammonium sulfate), other 7 SOE systems had almost no significant effect on the T7 phage titer, and the recovery rate of the phage was as high as 100%. Obviously, tert‐butanol in system 3 had a significant effect on the T7 phage titer, and the recovery rate of the phage was only 0.1%. This organic solvent can be used to kill T7 phage if necessary.

In addition, the removal of cells/cell debris and proteins in different SOE systems were also investigated. The experimental results showed that the system 2, 4, 5, 6, and 8 had a poor removal efficiency for either cells and cell debris (system 2, 4, and 5) or proteins (system 6 and 8). In particular, no removal effects were observed on cells in system 2 and 4, or proteins in system 8 as shown in Figure 5.

Comparatively, the system 1 or 7 composed of ethyl acetate /ammonium sulfate or ammonium citrate was the appropriate SOE system for recovery of phage and κ‐carrageenase as well as removal of cells/cell debris and proteins. Additionally, considering ammonium sulfate as salting‐out agent for the target enzyme in the bottom phase, the SOE system of ethyl acetate/ammonium sulfate was selected for further investigation.

### 3.3 Optimization of SOE system

**FIGURE 6:**
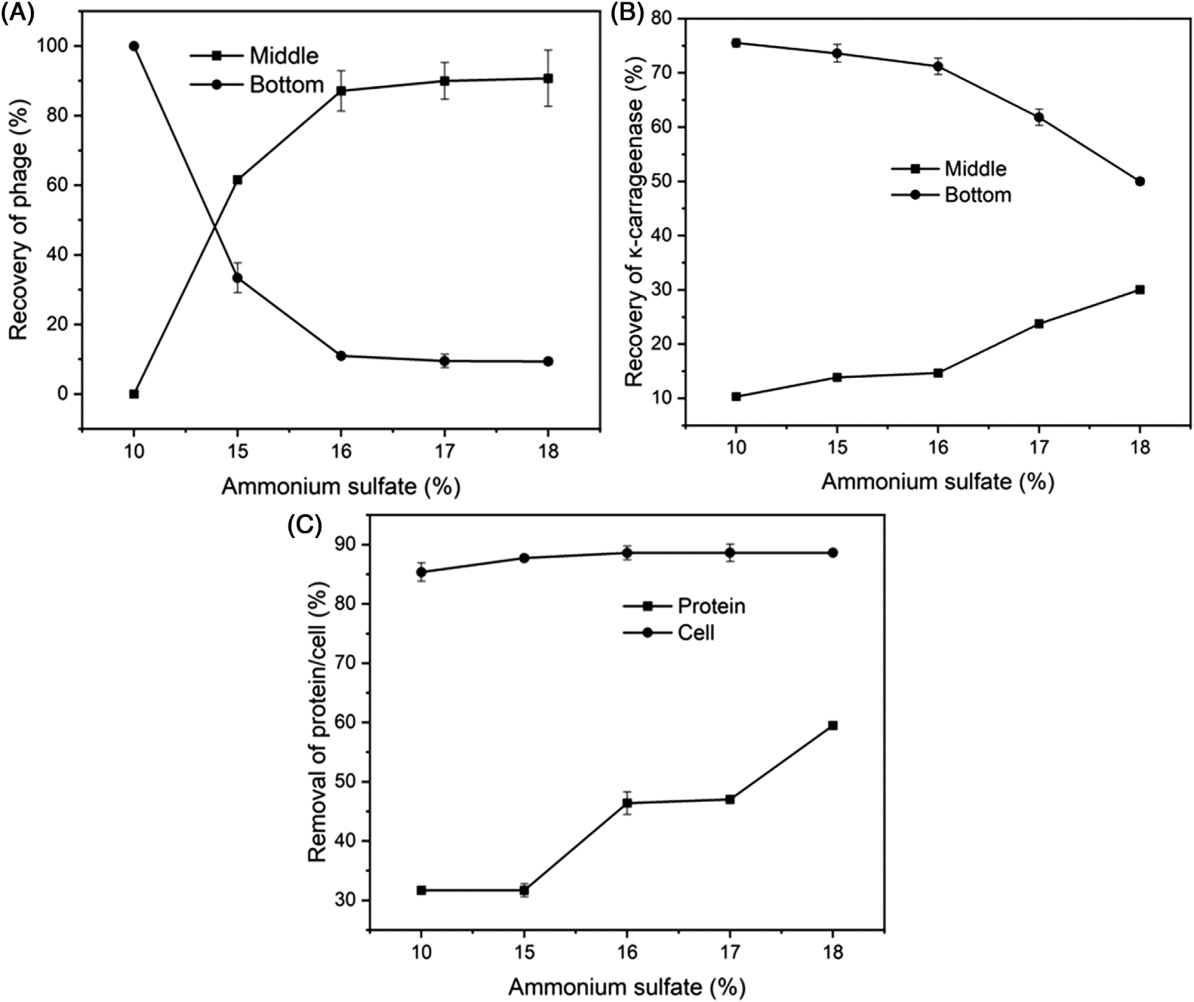
Effects of ammonium sulfate on phage (A) and κ‐carragenase (B) recovery, cells/cell debris and protein removal (C) in salting‐out extraction system composed of ethyl acetate/ammonium sulfate.

In the SOE system composed of ammonium sulfate and ethyl acetate, the mass fractions of ethyl acetate were fixed at 20%, and different mass fractions of ammonium sulfate exhibited a significant influence on the partitioning of the κ‐carrageenase and phage as shown in Figure 6A,B. When the mass fraction of ammonium sulfate was 10%, 100% of phage and 75.5% κ‐carrageenase accumulated in the bottom phase. As the mass fraction of salt increased, phages were transferred from the bottom phase to the middle phase. When the salt concentration increased to 16% (w/w), the recovery rate of phages in the middle phase was 87.1%, and 71.2% of κ‐carrageenase was obtained in the bottom phase. Upon further increasing the mass fraction of salt, κ‐carrageenase also began to transfer from the bottom phase to the middle phase, and the recovery of phages remained almost unchanged in the middle phase. This was likely attributed to the “salting out” effect (precipitation) of ammonium sulfate on the enzyme [36] because of the increase in ionic strength as the salt concentration increases.

On the other hand, as the mass fraction of ammonium sulfate increased, the cells/cell debris and proteins in the bottom phase were transferred to the middle phase as shown in Figure 6C. When the mass fraction of ammonium sulfate was 16%, 46.4% of total protein and 88.6% of cells/cell debris were aggregated into the middle phase. In order to reduce the accumulation of cells and cell debris in the middle phase, the phage lysate can be pretreated (e.g., centrifugation or microfiltration) before SOE [27].

When the mass fraction of ammonium sulfate was fixed at 16%, recovery rates of κ‐carrageenase and phages, as well as removal of cells/cell debris and proteins, were almost unchanged regardless of the change of mass fraction of ethyl acetate (data not shown). Therefore, the optimized SOE system was determined to be 16% ammonium sulfate and 20% ethyl acetate (w/w), in which 87.1% of the phage was distributed at the middle phase and 71.2% of κ‐carrageenase in the bottom phases, respectively.

### 3.4 Comparison of two separation processes

**FIGURE 7:**
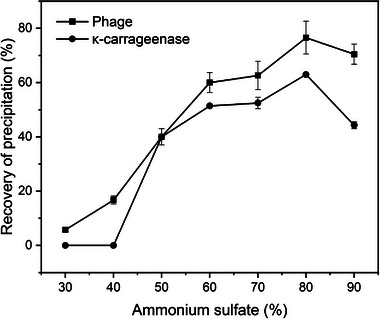
Recovery rates of phage and κ‐carragenase by salting out of ammonium sulfate.

κ‐Carragenase in cell lysates is usually salted out by ammonium sulfate [10]. In order to determine the optimal concentration of ammonium sulfate for salting out of the enzyme, phage lysate was obtained under the optimal conditions for phage lysis and used to separate κ‐carrageenase as well as phage as shown in Figure 7. When the saturation of ammonium sulfate was 80%, and the recovery rates of the phage and κ‐carrageenase were 76.5% and 61.4%, respectively. Obviously, it would be very difficult to separate κ‐carrageenase from phage if no separation units were used before salting out. When SOE was carried out after phage lysis, κ‐carrageenase would be the target product in salting out from the salt‐rich bottom phase after SOE. As an impurity, T7 phage can be killed by tert‐butanol. The presence of tert‐butanol will be favorable for the freeze drying of κ‐carrageenase in the further step [37].

Compared with the traditional separation route, that is, route A: ultrasonication+ centrifugation + salting out, the recovery of κ‐carrageenase has certain characteristics by means of a new separation route, that is, phage lysis + SOE + salting out (route B). The results are shown in Table 1.

**TABLE 1:** Comparison of κ‐carrageenase recovery by two separation processes.

Unit operation	Separation index	Process A^a^	Process B^b^
Cell disruption	Total enzyme activity (U)	10.10 ± 0.01	9.23 ± 0.00
	Total proteins (mg)	2.29 ± 0.05	2.27 ± 0.11
	Total specific activity (U/mg)	4.41 ± 0.18	4.10 ± 0.48
Centrifugation (A) or SOE (B)	Total enzyme activity (U)	10.10 ± 0.01	6.63 ± 0.17
	Total proteins (mg)	2.29 ± 0.05	1.20 ± 0.23
	Total specific activity (U/mg)	4.41 ± 0.18	5.53 ± 0.43
Salting out	Total enzyme activity (U)	5.76 ± 0.16	3.70 ± 0.08
	Total proteins (mg)	0.56 ± 0.04	0.33 ± 0.05
	Total specific activity (U/mg)	10.28 ± 0.42	11.21 ± 1.19
	Purification fold	2.33 ± 0.15	2.76 ± 0.25
	Recovery (%)	57.0 ± 0.16	40.1 ± 0.08

^a^ A: Ultrasonication + centrifugation + salting out.

^b^ B: Phage lysis + SOE + salting out.

From the view of cell disruption, the release rates of κ‐carrageenase by means of phage lysis corresponds to 91.4% of the release by ultrasonication, and 99.1% for total proteins. Two kinds of cell disruption methods are comparable. In another word, phage lysis can replace ultrasonication for cell disruption.

The final recovery rates of κ‐carrageenase using the route A in this research and the previous report [10] were 57% and 65.5%, respectively. The difference in recovery was due to the lower total enzyme activity before salting out in this research. Comparison of two separation routes A and B, the final recovery rates of κ‐carrageenase by salting out of ammonium sulfate existed a difference (57.0% vs. 40.1%), and the purification folds exhibited reverse tendency, that is, 2.33 (A) versus 2.76 (B) as shown in Table 1. This was attributed to SOE, by which another product (T7 phage) can be obtained simultaneously. Phage can offset the economic loss of the enzyme due to a lower recovery in the route B.

Considering economic factors, the route B has some advantages over the route A. For examples, in the route A, ultrasonication was used for cell disruption and high‐speed centrifugation was used for removal of cell debris, which need specific and precious apparatuses with high operation and safeguard costs. On the contrary, in the route B, phage lysis and SOE can be performed under simple and mild conditions with low cost. Additionally, the new separation route (B) is easy to scale up.

## 4 CONCLUDING REMARKS

In this study, T7 phage was used for the first time as a cell disruption tool to lyse the cell wall of *E. coli*. The results showed that T7 phage efficiently lysed *E. coli* at MOI = 1, with a cell disruption rate up to 99.9% and a release rate of intracellular products up to 91.4%. The phage and κ‐carrageenase were distributed in the intermediate and bottom phases using an SOE system composed of 16% ammonium sulfate and 20% ethyl acetate (w/w), and the recovery rates of the phage and κ‐carrageenase were 87.1% and 71.2%, respectively. Subsequently, the κ‐carrageenase in the bottom phase could be salted out by ammonium sulfate at 80% saturation, and the recovery rate was 40.1%. Compared with traditional cell disruption techniques, phage lysis coupled with SOE can efficiently lyse *E. coli* and has the advantages of low cost and easy operation. This novel separation method is of high relevance for the genetic engineering products by providing viable alternatives to the classical cell disruption and separation.

## AUTHOR CONTRIBUTIONS

Da Chen: Conceptualization, methodology, investigation, formal analysis, writing – original draft, and writing‐review & editing. Yue‐Sheng Dong: Writing‐review & editing. Yong‐Ming Bao: Writing–review & editing and resources. Zhi‐Long Xiu: Conceptualization, writing‐review & editing, methodology, supervision, and funding acquisition.

## CONFLICT OF INTEREST STATEMENT

We hereby certify that this paper is original. No conflict of interest exists in the paper.
